# Electroconvulsive therapy for Parkinson's disease‐related apathy: A case report

**DOI:** 10.1002/pcn5.70192

**Published:** 2025-08-21

**Authors:** Kiyori Yamanaka, Ryo Mizui, Yuki Noriyama, Yuya Honda, Ryohei Takada, Takashi Okada

**Affiliations:** ^1^ Department of Psychiatry Nara Medical University School of Medicine Kashihara Japan

**Keywords:** apathy, modified electroconvulsive therapy (m‐ECT), Parkinson's disease

## Abstract

**Background:**

Nonmotor symptoms are a critical focus in the management of Parkinson's disease (PD). Apathy is defined as a quantitative reduction in goal‐directed activity, characterized by diminished initiative, interest, and emotional expression or responsiveness. It affects approximately 40% of patients with PD, severely impairing daily functioning and quality of life. Although electroconvulsive therapy (ECT) is well established for alleviating depressive symptoms in patients with PD, its efficacy in treating apathy remains largely unexplored.

**Case Presentation:**

We report the case of a 54‐year‐old man diagnosed with PD at age 49, whose motor symptoms were well controlled with optimized pharmacotherapy. However, at age 53, he developed profound apathy, characterized by a notable reduction in spontaneous movement and emotional expression. Given the lack of response to pharmacological adjustments, including a trial of venlafaxine, modified ECT (m‐ECT) was initiated. The patient underwent 10 m‐ECT sessions over 5 weeks (twice weekly) using propofol and succinylcholine. Following treatment, his apathy scale score improved substantially from 32 to 12, with no adverse effects on cognitive function.

**Conclusion:**

This case highlights the potential efficacy of ECT for PD‐related apathy. Further research is needed to elucidate its underlying mechanisms and assess the long‐term outcomes of ECT in managing nonmotor symptoms in PD.

## BACKGROUND

Apathy is defined as a quantitative reduction in goal‐directed activity, characterized by diminished initiative, interest, and emotional expression or responsiveness.^1^ It is a debilitating syndrome commonly associated with neurological and psychiatric disorders such as dementia, traumatic brain injury, major depression, and schizophrenia.

Among various neurotransmitter systems implicated in the pathophysiology of apathy, the dopaminergic system has been most extensively studied,^2^ and in fact, approximately 40% of patients with Parkinson's disease (PD) are diagnosed with apathy.^3^, ^4^ PD is characterized by degeneration of dopaminergic neurons in the substantia nigra, leading to dopaminergic dysfunction in the anterior cingulate cortex and ventral striatum, which includes the nucleus accumbens, which plays a crucial role in the development of apathy. Apathy is a characteristic nonmotor symptom of PD and may be associated with impaired daily functioning, reduced treatment response, poor clinical outcomes, and diminished quality of life.^5^

Pharmacological approaches for apathy in patients with PD have included dopamine agonists, such as methylphenidate, and cholinesterase inhibitors. However, pharmacological treatments are sometimes discontinued due to adverse effects or limited efficacy. As alternatives, non‐pharmacological approaches, interventions such as exercise and cognitive training may prove beneficial.^4^, ^6^ In addition, modified electroconvulsive therapy (m‐ECT) may modulate dopaminergic neurotransmission and can, thus, be a viable treatment option for psychiatric symptoms comorbid with PD. In fact, case reports suggest that ECT is effective for treating both motor and nonmotor symptoms, including depression and psychosis, in patients with PD without worsening cognitive function.^7^ However, to our knowledge, its effectiveness in treating apathy without comorbid psychotic symptoms as a primary target symptom of PD has not yet been reported.

Here, we report a case of a 54‐year‐old man with PD and apathy whose condition improved with ECT. This finding supports the potential efficacy of ECT for PD‐related apathy and underscores the need for further research to elucidate its mechanisms and long‐term effects.

## CASE PRESENTATION

A 54‐year‐old man was diagnosed with PD at age 49 and had been receiving pharmacological treatment from a neurologist at our hospital. Imaging studies performed at age 51 supported the diagnosis of PD. ^123^I‐FP‐CIT SPECT showed decreased tracer uptake in the bilateral striatum, with specific binding ratios of 4.44 on the right and 3.82 on the left (average 4.13), and an asymmetry index of 15.0. The *Z*‐scores were –3.57 on the right and –3.86 on the left. ^123^I‐MIBG myocardial scintigraphy revealed reduced cardiac sympathetic innervation, with heart‐to‐mediastinum ratios of 2.11 (early) and 1.46 (delayed), and a washout rate of 30.8%. Around September 10 of the year before the index year (X‐1), at age 53, he began experiencing lower limb weakness. On September 14, he was diagnosed with an exacerbation of parkinsonian symptoms and was admitted to the neurology department the same day. Required revisions were made to the prescribed medication in the outpatient unit, leading to stabilization and partial improvement of his motor symptoms. Consequently, on October 31, X‐1, he was transferred to another hospital for rehabilitation.

**Figure 1:**
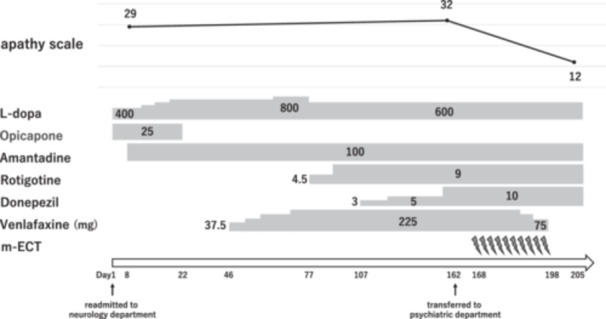
Treatment and clinical course. Timeline of medication adjustments during the patient's treatment course. The figure illustrates sequential changes in antiparkinsonian medications, antidepressants, and donepezil dosing from admission through the entire hospitalization, providing an overview of the treatment regimen. The apathy scale values at different time points are also shown. m‐ECT, modified electroconvulsive therapy.

In November X‐1, the patient developed an unexplained decline in motivation, which interfered with his rehabilitation progress. His spontaneous motor activity gradually decreased; eventually, he required total assistance. On December 19, X‐1 (Day 1), he was readmitted to our neurology department. Since there was no worsening of parkinsonism, he was diagnosed with apathy associated with PD. At that time, his assessment revealed an apathy scale (AS)^8^ score of 29, indicating severe apathy (Table 1). In contrast, his Hamilton Depression Rating Scale (HAM‐D) score was 12 for diminished motivation, with no prominent depressed mood. He was able to respond to simple instructions; hence, we determined that he was not in a stupor. There were no findings suggestive of catatonia, such as catalepsy or echolalia. Despite increasing the levodopa dosage from 400 to 800 mg/day and adding amantadine (100 mg/day), rotigotine (9 mg/day), and donepezil (10 mg/day), there was no improvement in apathy. Therefore, a psychiatric consultation was requested. Given the patient's limited range of expressive language observed, concerns about a possible underlying depressive disorder prompted the administration of venlafaxine (225 mg/day). However, neither an improvement in apathy nor a reduction in symptom exacerbation was observed. Consequently, the clinical presentation was reassessed as apathy, primarily characterized by diminished emotional expression and reduced spontaneity. Therefore, non‐pharmacological treatment options were considered. The detailed course of medication adjustments is shown in Figure 1.

**Table 1:** Apathy scale.

		Day 1	Day 162	Day 205
1	Are you interested in learning new things?	2	3	2
2	Does anything interest you?	2	3	2
3	Are you concerned about your condition?	2	1	0
4	Do you put much effort into things?	2	2	1
5	Are you always looking for something to do?	2	3	1
6	Do you have plans and goals for the future?	2	3	1
7	Do you have motivation?	2	3	1
8	Do you have the energy for daily activities?	2	3	2
9	Does someone have to tell you what to do each day?	2	2	0
10	Are you indifferent to things?	2	2	0
11	Are you unconcerned with many things?	3	2	0
12	Do you need a push to get started on things?	2	2	0
13	Are you neither happy nor sad, just in between?	2	1	1
14	Would you consider yourself apathetic?	2	2	1
	Total	29	32	12

*Note*: Scoring—for questions 1–8, not at all = 3 points; slightly = 2; some = 1; a lot = 0. For questions 9–14, not at all = 0; slightly = 1; some = 2; a lot = 3.

Deep brain stimulation was considered as a treatment option; however, the patient and his family were reluctant to undergo surgical intervention, and therefore, m‐ECT was selected based on the results of two previous case reports.^9^, ^10^ Blood tests, cranial magnetic resonance imaging, and electroencephalography revealed no abnormalities, and contraindications to m‐ECT were ruled out. After providing a thorough explanation and obtaining informed consent from both the patient and his family, he was transferred to our department on May 28, year X (Day 162), and m‐ECT was initiated. At the time of transfer, his evaluation revealed an AS score of 32 and a HAM‐D score of 12. Although the Mini‐Mental State Examination (MMSE) was considered, it could not be administered due to the patient's lack of motivation. m‐ECT was performed twice weekly for 10 sessions over a 5‐week period, using propofol for anesthesia and succinylcholine as a muscle relaxant. Sessions 1–5 were bilateral, with the electrical charge adjusted to achieve a seizure threshold of 126 mC and a stimulation duration of 7.0 s. In Session 6, due to insufficient seizure activity, the electrical charge was increased to 252 mC for the same duration, followed by bilateral electrical intensity. Further adjustments were made as needed and maintained until Session 10.

The first noticeable improvement in facial expression was observed on Day 175 (after the third ECT), although the patient remained largely unaware of any symptomatic changes. By Day 184 (after the sixth ECT), he began to smile and actively engage in rehabilitation. By Day 191 (after the eighth ECT), spontaneous speech increased, and he engaged in watching TV and reading magazines. On Day 197 (after the 10th ECT), he reported feeling substantially better. At the time of discharge from the hospital on Day 205, his AS score had improved from 32 to 12 (Table 1), and his HAM‐D score was 5. The Hoehn and Yahr scale was Stage III, both before and after ECT, with no marked change. No side effects, such as amnesia, cognitive decline, or manic symptoms, were observed following m‐ECT. This assessment was also supported by impressions shared by the patient's family during conversations with him.

After transfer to our department, venlafaxine was discontinued, but no adjustments were made to antiparkinsonian medications, which had minimal influence on the observed improvement.

## DISCUSSION

Recently, addressing nonmotor symptoms has become pivotal in PD management. Among these symptoms, depression and apathy are the most prevalent, affecting approximately 35% and 40% of patients, respectively.^4^, ^11^ To better characterize mood disturbances in patients with PD, efforts have been made to distinguish apathy from depression.^12^, ^13^ Because mood symptoms can be easily masked in these patients, caution is required when ruling out depression, even when only partial depressive symptoms are present.^14^ In this case, given the patient's limited verbal expression, the possibility of an underlying depression could not be ruled out. However, depressive mood was absent, and apathy was identified as the primary pathology, characterized by diminished emotional expressiveness and spontaneity.

Although m‐ECT is well known for its efficacy in alleviating depressive symptoms, its effects on apathy remain poorly understood. Apathy has been linked to dysfunction in the dopaminergic system, particularly in the ventral striatum.^2^ Neuroplastic changes induced by ECT have been extensively studied, particularly in the dentate gyrus of the hippocampus.^15^, ^16^, ^17^ In addition to these effects, animal models show that ECT enhances dopaminergic function, increasing D1 and D3 receptor binding in the striatum of mice^18^ and enhance D1 receptor and vesicular monoamine transporter Type 2 binding in primates.^19^ Furthermore, a case report described the effectiveness of ECT for apathy in a patient with PD, whose dopamine transporter single‐photon emission computed tomography showed decreased uptake in the right caudate.^9^ These findings suggest that the therapeutic effects of ECT on apathy may be mediated by its ability to modulate dopaminergic transmission, especially in the striatum. However, to our knowledge, only one case report has described the use of ECT for apathy in PD: this included psychotic features such as delusions.^9^ It is important to emphasize that our patient did not exhibit psychiatric or neuropsychiatric symptoms. The simplicity of the clinical presentation highlights the effectiveness of ECT in patients with PD and distinctive apathy profiles.

## LIMITATIONS

This case report has several limitations. First, standardized cognitive assessments were not conducted, making it difficult to evaluate subtle cognitive changes before and after m‐ECT. Second, while depressive symptoms were not observed, the patient's limited verbal expression hindered definitive differentiation between apathy and subclinical depression—an acknowledged diagnostic challenge in PD. Third, no follow‐up neuroimaging was performed to assess functional changes associated with treatment. These limitations should be considered when interpreting the present findings and their generalizability.

## CONCLUSION

This case report suggests that ECT may be an effective treatment option for apathy in PD. The patient's apathy persisted despite optimal management of motor symptoms and a trial of pharmacological treatment. Following a course of ECT, he exhibited marked improvement in apathy without cognitive deterioration. These findings support the potential role of ECT in addressing nonmotor symptoms, particularly apathy, which negatively impacts daily functioning and quality of life in patients with PD. Further studies are warranted to clarify the underlying mechanisms and assess the long‐term outcomes of ECT in this population.

## AUTHOR CONTRIBUTIONS

Kiyori Yamanaka, Yuki Noriyama, and Ryo Mizui were involved in the treatment. Kiyori Yamanaka, Yuki Noriyama, and Ryohei Takada prepared the manuscript. Ryo Mizui, Ryohei Takada, and Takashi Okada critiqued and revised the manuscript. All authors read and approved the final version.

## CONFLICT OF INTEREST STATEMENT

The authors declare no conflicts of interest.

## ETHICS APPROVAL STATEMENT

N/A.

## PATIENT CONSENT STATEMENT

Written informed consent was obtained from the patient for publication of the case report.

## CLINICAL TRIAL REGISTRATION

N/A.

## Data Availability

Data sharing is not applicable to this article as no datasets were generated or analyzed during the current study.
